# Time-restricted feeding affects colonic nutrient substrates and modulates the diurnal fluctuation of microbiota in pigs

**DOI:** 10.3389/fmicb.2023.1162482

**Published:** 2023-05-19

**Authors:** Hongyu Wang, Qiuke Li, Rongying Xu, Yong Su, Weiyun Zhu

**Affiliations:** ^1^Laboratory of Gastrointestinal Microbiology, Jiangsu Key Laboratory of Gastrointestinal Nutrition and Animal Health, College of Animal Science and Technology, Nanjing Agricultural University, Nanjing, China; ^2^National Center for International Research on Animal Gut Nutrition, Nanjing Agricultural University, Nanjing, China

**Keywords:** gut microbiota, growing pigs, time-restricted feeding, microbial rhythmicity, 16S rRNA sequencing

## Abstract

**Introduction:**

Studies demonstrate that time-restricted feeding (TRF) can regulate gut microbiota composition. However, it is unclear whether TRF could affect the gut microbial rhythmicity in growing pigs. Therefore, the present study aimed to explore the effects of TRF on the dynamic fluctuation of the gut microbiota.

**Methods:**

A total of 10 healthy growing pigs equipped with T cannula were employed. Pigs were randomly allotted to the free access (FA) and the TRF groups with 5 replicates (1 pig/replicates). Pigs in the FA group were fed free access during the whole experimental period, whereas pigs in the TRF group were fed free access three times per day within limited times (7:00–8:00, 12:00–13:00, 17:00–18:00). The experiment lasted for 15 days, at 06:00 a.m. of the day 16, colonic digesta were collected at a 6-h interval for consecutive 24 h marked as T06 (06:00), T12 (12:00), T18 (18:00), T24 (24:00), T30 (06:00), respectively.

**Results:**

Results showed that TRF altered the distribution of feed intake without changing the total feed intake within a day (*p* = 0.870). TRF decreased the overall concentration of colonic cellulose and altered their oscillating patterns. All alpha-diversity indexes of different time points showed significant differences regardless of feeding pattern with a trough at T18 or T24. TRF shifted the trough of the alpha-diversity index Simpson and Invsimpson. TRF lost the rhythmicity of *Prevotellaceae*, *Ruminococcaceae*, *Bacteroidales*_S24-7_group, and *Peptococcaceae* and gained the rhythmicity of *Pasteurellaceae*, *Clostridiaceae*_1, *Veillonellaceae*, and *Peptostreptococcaceae*. Also, TRF altered the interaction pattern by increasing the microbes involved in the co-occurrence network and their crosstalk, especially at T24. Interestingly, the microbial variation at T24 could largely explained by colonic substrates starch (R^2^ = 0.369; *p* = 0.001), cellulose (R^2^ = 0.235; *p* = 0.009) and NH4-N (R^2^ = 0.489; *p* = 0.001).

**Conclusion:**

In conclusion, TRF has changed the concentrates of cellulose and the relative abundance of specific microbes and certain microbial metabolites. In addition, TRF has more powerful effects on the fluctuation modes of these nutrient substrates, microbes, and metabolites by shifting their peaks or troughs. This knowledge facilitates the development of precision regulation targeting gut microbial rhythmicity.

## 1. Introduction

The mammalian digestive tract harbors a variety of microorganisms with populations over 100 trillion (Qin et al., 2012). These microorganisms play crucial roles in maintaining normal physiology, nutrition, metabolism, and health (Kim et al., 2012; Sommer and Backhed, 2013; Knecht et al., 2020). Studies have revealed that the microbiota in the mammal gastro-intestine such as mice and human undergoes robust diurnal oscillation within 1 day in abundance, function, and biogeography defined as the gut microbial rhythmicity (Thaiss et al., 2014; Leone et al., 2015; Thaiss et al., 2016; Zhang et al., 2021). Despite that the possible mechanism underlying the formation of the gut microbiota rhythmicity is not fully understood yet, accumulating evidence confirms that the normal fluctuation of the gut microbiota has more profound implications on host health, physiology, and immunity (Shao et al., 2018; Reitmeier et al., 2020; Allaband et al., 2021). The oscillation of the gut microbiota is susceptible to many factors derived from the host, environment, and diet, including but not limited to genetics, gender, age, physiology, and feed (Wang et al., 2022). The host circadian clock, which synchronizes the normal rhythms of physiological activities including eating, sleeping, and the secretion of hormones with the diurnal variation in the external environment, is undoubtedly an essential factor that affects the gut microbiota rhythmicity (Vandewalle et al., 2009; Liang et al., 2015; Thaiss et al., 2016). However, the loss of the gut microbiota rhythmicity induced by the depletion of the core circadian clock gene was rescued by the TRF feeding mode (Thaiss et al., 2016). These results unravel the vital role of nutrition in the maintenance of the normal fluctuation of the gut microbiota (Xiao et al., 2016). To date, most of the data on gut microbial rhythmicity have abstained from rodent models. However, there are considerable species differences in microbial composition and function between humans and rats (Xiao et al., 2016). Moreover, samples from humans are limited by fixed sampling times, ethnic problems, and are easily contaminated. Metagenomic sequencing data revealed that the pig gut microbiome catalog has 96% of the functional pathways associated with the human gut microbiome catalog (Xiao et al., 2016). Considering the closer relationship and higher similarities concerning the body size, anatomical structures, normal physiology, eating habits, microbial composition, and function, the pig model may be one of the most suitable animal models for human studies (Simon and Maibach, 2000; Guigo et al., 2003; Bassols et al., 2014; Xiao et al., 2016).

TRF is generally becoming a popular therapy as it can effectively improve chronically metabolic syndromes including obesity, hypertension, diabetes, hyperlipidemia, and cardiovascular and cerebrovascular diseases induced by overnutrition, unhealthy living habits, jet lag, and time shift work in modern society (Zarrinpar et al., 2014; Zeb et al., 2020a). Moreover, these metabolic syndromes have unavoidable associations with dysbiosis and arrthythmicity of the gut microbiota (Turnbaugh et al., 2006; Bouter et al., 2017; Reitmeier et al., 2020). Convincing evidence suggests that dietary factors such as diet composition, nutrient level, probiotic additives, and eating patterns might affect the diurnal fluctuation of the gut microbiota (Zarrinpar et al., 2014; Wang et al., 2020; Sun et al., 2021). Of note, as the essential nutrients for living organisms, carbon and nitrogen sources (more specifically, the level of starch, cellulose, and the crude protein in the diet) play a vital role in the regulation of microbial composition (Gao et al., 2019; Liu et al., 2020; Li et al., 2021). Furthermore, researchers have shown that TRF patterns alter the nutritional profile in the gut and these microbial shifts are associated with the nutrients in pigs (Zeb et al., 2020b; Wang et al., 2021). Thus, we speculated the shift feeding mode could alter the dynamic homeostasis of gut microbiota by regulating the fluctuation of the nutrient substrates. In the present study, we aimed to explore whether the TRF pattern affects the fluctuation of the gut microbes and their possible associations with the nutrient substrates.

## 2. Materials and methods

### 2.1. Ethnic statement

The experiment was authorized by the Animal Care and Use Committee of Nanjing Agricultural University (Nanjing, Jiangsu Province, China; SYXK2019-0066). All procedures related to the experimental animal were carried out by the Experimental Animal Care and Use Guidelines of China (EACUGC2018-01).

### 2.2. Experimental design and sampling

**Figure 1:**
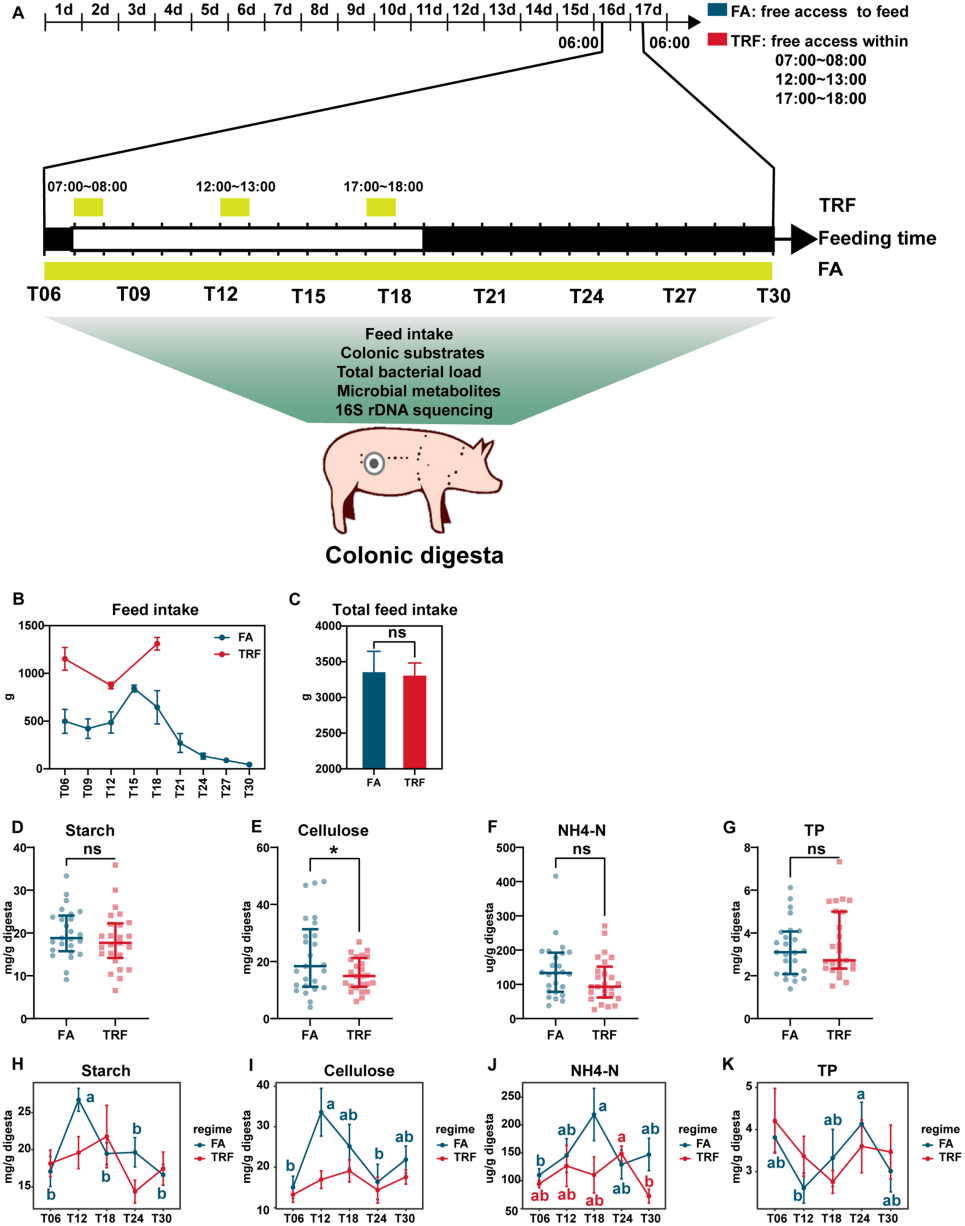
Effect of TRF on feed intake and the colonic nutrient substrates of growing pigs. **(A)** Flowchart of the experiment. **(B)** Dynamic changes in feed intake during each sampling interval within a day. **(C)** Effect of TRF on the total feed intake of growing pigs in a day. The difference in total feed intake between the FA group and the TRF group was determined by an unpaired two-tailed t-test. Ns represents *p* > 0.05. **(D–G)** Effect of TRF on the overall nutrient substrates starch **(D)**, cellulose **(E)**, NH4-N **(F)**, and TP **(G)** in the colon digesta. The difference in starch, cellulose, and NH4-N between the FA group and the TRF group was determined by an unpaired two-tailed t-test. Whereas the difference in TP between the FA group and the TRF group was determined by a non-parametric test. Ns represents *p* > 0.05, whereas * denotes a significant difference with a *p* < 0.05. **(H–K)** Effect of TRF on the dynamic changes of nutrient substrates starch **(H)**, cellulose **(I)**, NH4-N **(J)**, and TP **(K)** in the colon digesta. Differences in nutrient substrates at the different time points of each group were identified by one-way analysis of variance, followed by Bonferroni-based multiple comparisons. NH4-N, Ammonia nitrogen; TP, total protein.

A total of 10 healthy growing pigs (Duroc × Landrace × Large White, 110 days) with an average body weight of 56.27 ± 1.56 kg were employed in the experiment. Pigs were fitted with T-cannula at the proximal colon. Pigs were randomly allotted to the FA group and TRF group with 5 replicates (1 pig/group) according to body weight. All pigs were fed with the same commercial pellet for growing pigs. Pigs in the FA group were fed free access during the whole experimental period, whereas pigs in the TRF group were fed three times per day within limited times (7:00–8:00, 12:00–13:00, 17:00–18:00) to mimic the three-meal pattern in modern society (Figure 1A). During feeding time, pigs were fed free access. The experiment lasted for 15 days. At 06:00 am of day 16, colonic digesta were collected at a 3-h interval for consecutive 24 h marked as T06 (06:00), T12 (12:00), T18 (18:00), T24 (24:00), and T30 (06:00), respectively. These digesta samples were stored in liquid nitrogen for further analysis of microbiota, metabolites, and nutrient substrates. Feed intake during each sample interval was also recorded.

### 2.3. The determination of the colonic nutrient substrates

The starch concentration in the colonic digesta was measured by a commercial Starch Detection Kit (BC0700; Solaibao, Beijing, China) according to the instructions of the manufacturer. Firstly, 80% (v/v) ethanol was used to remove soluble sugars in the samples. Then the retained samples were gelatinized in a 100°C water bath. The gelatinized starch was hydrolyzed by 52% (v/v) perchloric acid. The free glucose obtained in the hydrolysate was measured through anthrone colorimetry referring to the previous reference (Zhuang et al., 2019).

Cellulose concentration was measured by a commercial Cellulose Detection Kit (BC4280; Solaibao, Beijing, China). Shortly, cell wall materials were extracted from the digesta sample after removing the soluble impurities using 80% (v/v) ethanol and acetone. Then, the obtained cell wall materials were hydrolyzed to free glucose by sulfuric acid. The measurement of free glucose was similar to that of the starch measurement (Zhuang et al., 2019).

The concentration of total protein (TP) was measured by a commercial Protein Detection Kit (PC0010; Solaibao, Beijing, China) using the Coomassie brilliant blue G-250 method. Both the digesta sample and standard were dissolved in saline solution. After thoroughly mixing, the absorbances were recorded at a wavelength of 595 nm. All procedures were followed to the instructions in a previous reference (Li et al., 2019).

NH4-N was determined by the colorimetric method referring to the previous method (Wang et al., 2021). In short, digesta sample was dissolved in 0.2 mol/L HCl solution. The supernatant was then thoroughly mixed with sodium hypochlorite-sodium hydroxide solution and 0.08% (w/v) sodium nitroprusside sodium salicylate. The absorbance of the reaction mixture was measured at a wavelength of 700 nm.

### 2.4. DNA extraction, 16S rRNA amplification, and sequencing

Microbial DNA from digesta was extracted by the cetrimonium bromide method (Dai et al., 2010). About 0.3 g digesta sample was repeatedly washed and centrifuged with saline to remove impurities. After removing the supernatant, the sediment was resuspended in the CTAB solution. The mixture then underwent a vigorous vortex to release DNA from bacterial cells. After removing the RNA in the mixture using RNase, DNA was purified by repeated extraction of phenol/chloroform/isoamyl alcohol and ethanol precipitation. The DNA sample was resuspended in TE buffer for quality control and further analysis. The obtained DNA sample was amplified using a universal primer with the unique barcode [forward primer (5′-CCTAYGGGRBGCASCAG-3′) and reverse primer (5′-GGACTACNNGGGTATCTAAT-3′)]. NEB Next®Ultra™DNA Library Prep Kit for Illumina (NEB, United States) was used to establish the sequencing libraries referring to the manufacturer’s instruction (Cronin et al., 2018; Kers et al., 2018). Sequencing was finished on an Illumina MiSeq platform and paired-end reads with 250/300 bp were finally generated.

### 2.5. Bioinformatic analysis

Paired-end reads were merged according to overlaps using VSEARCH (v2.20.1). Merged reads with an error threshold of more than 0.01 were removed after trimming specific primers and barcodes. Clean reads were denoised after the disposal of low-abundance noise with a miniquesize of 8 using the UNOISE3 in USEARCH (v10.0.240) to obtain amplicon sequence variants (ASVs). Chimeras were removed by aligning against the Ribosomal Database Project (RDP; v1.8). Feature ASVs were produced using USEARCH (v10.0.240) and then annotated by aligning against the RDP database (v1.6) using VSEARCH (v2.20.1) with a confidence of 0.97. Read counts were normalized using an R package vegan. Alpha-diversity indices and the rarefaction curve of each group were calculated by USEARCH (v10.0.240). Principal Coordinate Analysis (PCoA) analysis based on the Bray-Curtis distance was calculated. Further, the differences among each group were identified by PERMANOVA using the adonis function in the vegan package.

### 2.6. Determination of lactate and short-chain fatty acids

The concentration of lactate was determined with a commercial Lactate Assay Kit (Nanjing Jiancheng Institute of Biological Engineering, China) using the colorimetric method. All procedures strictly followed the instructions in the kit. In brief, about 0.1 g digesta samples were diluted in distilled water, thoroughly mixed, and then centrifuged. A total of 0.02 mL supernatant was mixed with 1 mL of enzyme working solution and 0.2 mL chromogenic agent. The mixture was incubated at 37°C for 10 min and immediately terminated with 2 mL of stop solution. The absorbance of the reaction solution was measured at the 530 nm wavelength. The concentration of lactate was calculated following a standard curve method.

The concentrations of short-chain fatty acids (acetate, propionate, isobutyrate, butyrate, isovalerate, and valerate) were measured by gas chromatography method using Agilent 7890A gas chromatograph (Agilent Technologies, Wilmington, 147 DE) as previously described (Wang et al., 2011). In brief, about 0.3 g of digesta sample was dissolved in the distilled water. The obtained supernatant, after vigorous vortex and centrifugation, was mixed with 25% (w/v) metaphosphoric acid to precipitate soluble proteins in it. SCFAs in the supernatant were then extracted by ether. The concentration of each SCFA was calculated based on the standard curve method.

### 2.7. Determination of biogenic amines

The level of biogenic amines, including methylamine, putrescine, tyramine, tryptamine, cadaverine, spermine, and spermidine, was measured using liquid chromatography referring to the previous reference (Yang et al., 2014). Shortly, impurities (protein, fat, and peptide) in the sample were gradually removed by trichloroacetic acid and n-hexane. Samples were then derivatized by the addition of sodium bicarbonate, dansyl chloride, and NaOH solution. The mixture was incubated at 60°C for 30 min. The derivatization reaction was terminated by the addition of an ammonia solution. For further analysis, derivatives were repeatedly extracted by ether and then resuspended in the acetonitrile solution. The concentration of each biogenic amine was calculated on the standard curve method.

### 2.8. Processing, statistical analysis, and data visualization

The overall differences in concentrations of nutrient substrates and microbial metabolites or relative abundance of specific microbes were calculated by merging all colonic digesta samples from different time points in the same group. Statistical differences of overall and each timepoint in total feed intake, the colonic nutrient substrates, alpha diversity indexes, microbes, and microbial metabolites were analyzed by SPSS software (IBM SPSS 23.0, SPSS Inc). For data that conformed to a normal distribution, a two-sided Student’s t-test was used, and for data that did not conform to a normal distribution, a non-parametric test was used. Differences in different time points of each group were analyzed *via* one-way analysis of variance, followed by Bonferroni-based multiple comparisons using SPSS software (IBM SPSS 23.0, SPSS Inc). The rhythmicity of alpha-diversity induces, microbes and microbial metabolites was assessed by a non-parametric JTK_circle R package (Thaiss et al., 2016). An index with a *P*_Adj_ < 0.05 was considered to have rhythmicity. The correlations between the microbes and the nutrient substrates were determined by Spearman’s correlation analysis. Microbial co-occurrence network based on ASVs level was conducted by the MENA pipeline (Deng et al., 2012). The co-occurrence networks were then visualized by the igraph package (version 1.2.4.1). Redundancy analysis (RDA) was used to explore the interpretations of nutrient substrates to the microbial composition. In all statistical analyses, *p*-values lower than 0.05 were considered significant unless otherwise specified.

## 3. Results

### 3.1. TRF altered the fluctuation of feed intake and the colonic substrates

TRF has changed the distribution of feed intake without changing the total feed intake (*p* = 0.870, Figures 1B,C). Under the FA condition, the feed intake peaked at ZT15 and reached a trough during the dark phase. Whereas in the TRF regime, feed intake peaked at T18. Overall, TRF significantly decreased the concentration of cellulose (*p* = 0.032, Figure 1E) but did not affect starch (*p* = 0.365, Figure 1D), NH4-N (*p* = 0.151, Figure 1F), and TP (*p* = 0.567, Figure 1G). Likewise, TRF has no significant effects on the concentrates of colonic nutrient substrates (starch, cellulose, NH4-N, and TP) at each sampling time point (Figures 1H,I). But TRF altered the oscillating pattern of nutrient substrates in the colon. Under the FA condition, the concentrations of starch and cellulose synchronously peaked at T12, whereas peaked at T18 in the TRF group (Figures 1H,I). NH4-N peaked at T18 in the FA group, whereas peaked at T24 in the TRF group (Figure 1J). TP reached a trough at T12 in the FA group, whereas reached a trough at T18 in the TRF group (Figure 1K).

### 3.2. TRF affected the gut microbiota dynamics of growing pigs

**Figure 2:**
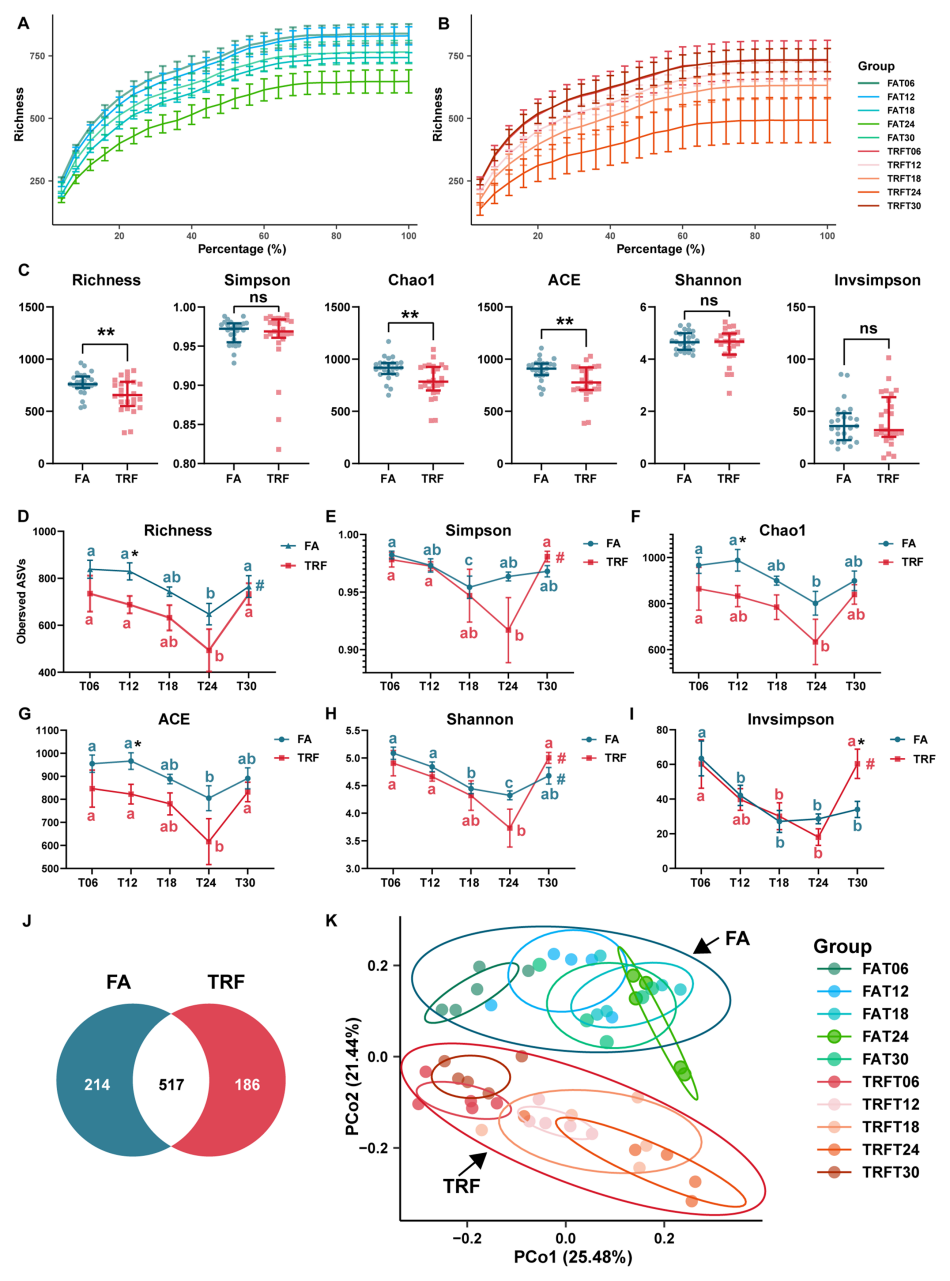
Effect of TRF on the microbial alpha diversity and the beta diversity in the colon community of growing pigs. **(A,B)** Rarefaction curves of different time points of the FA **(A)** and the TRF **(B)** groups. **(C)** Effect of TRF on the alpha-diversity Richness, Simpson, Chao1, ACE, Shannon, and Invsimpson of the colonic microbiota. The difference in Richness, Chao1, ACE, Shannon, and Invsimpson between the FA group and the TRF group was determined by an unpaired two-tailed t-test. Whereas the difference in Simpson between the FA group and the TRF group was determined by a non-parametric test. Ns represents *p* > 0.05, whereas ** denotes a significant difference with a *p* < 0.01. **(D–I)** Effect of TRF on the diurnal fluctuation in alpha-diversity Richness **(D)**, Simpson **(E)**, Chao1 **(F)**, ACE **(G)**, Shannon **(H)**, and Invsimpson **(I)** of the colonic microbial community. The difference in alpha diversity induced between the FA group and the TRF group at each time point was determined by an unpaired two-tailed *t*-test. * denotes a significant difference with a *p* < 0.05. Differences in alpha diversity at the different time points of each group were identified by a one-way analysis of variance, followed by Bonferroni-based multiple comparisons. Line points with different letters represent a significant difference within different time points. The rhythmicity of the alpha diversity of each group was determined by JTK_circle analysis. # denotes *P*_Adj_ < 0.05. **(J)** Venn diagram exhibiting the core ASVs identified from different groups. **(K)** Principal coordinates analysis (PCoA) plot based on the Bray–Curtis distance of different feeding modes at different sampling time points.

Up to 2,318,557 clean reads were generated from the 50 colonic digesta samples with an average of 46,371 ± 1,232 (mean ± standard error) clean reads per sample and an average clean read length of 413.74 ± 0.36 bp (Supplementary Table 1). Rarefaction curves of the FA group (Figure 2A) and TRF group (Figure 2B) tended to flatten out, indicating a reasonable amount of sequencing data. It is worth noting that TRF increased the variation in microbial richness compared to that of the FA group (Figures 2A,B). Alpha diversity Richness (*P*_Adj_ = 0.017, Figure 2D) of the FA group, Simpson (*P*_Adj_ = 0.017, Figure 2E) and Invsimpson (*P*_Adj_ = 0.017, Figure 2I) in the TRF group, and Shannon (FA: *P*_Adj_ = 0.024; TRF: *P*_Adj_ = 0.009. Figure 2H) in both groups had robust rhythmicity over time with a trough at T24. Of note, TRF has shifted the trough of alpha diversity Simpson and Invsimpson (Figures 2E,I). Interestingly, all alpha diversity indexes of different time points showed significant differences regardless of time (Figures 2D–I). Overall, TRF tended to decrease alpha diversity Richness (*p* = 0.007), chao1 (*p* = 0.004), and ACE (*p* = 0.003; Figure 2C). However, considering the sampling time points, TRF has limited effects on all alpha diversity at T06, T18, and T24. But, TRF significantly decreased the alpha diversity Richness (*p* = 0.026), chao1 (*p* = 0.045), and ACE (*p* = 0.033) at T12, whereas it increased Invsimpson at T30 (*p* = 0.033).

Using the denoise method, a total of 1,403 ASVs were identified from these clean reads. In total, *Firmicutes* (78.68% ± 1.91%) and *Bacteroidetes* (16.73% ± 1.79%) were the most dominant phylum in the colon microbial community of growing pigs (Supplementary Table 2). The relative abundance of the top 10 genera were *Streptococcus* (10.7 ± 1.36%), *Blautia* (5.92% ± 0.67%), *Ruminococcaceae_UCG-005* (5.43 ± 0.61%), *Prevotella_9* (4.66% ± 0.84%), *Alloprevotella* (4.35% ± 0.57%), *Subdoligranulum* (4.27% ± 0.35%), *Sarcina* (3.71% ± 1.09%), *Lactobacillus* (3.21% ± 0.68%), *Roseburia* (2.8% ± 0.28%), and *Anaerovibrio* (2.76% ± 0.42%; Supplementary Table 3). Core ASVs were defined as ASVs that were present in no less than 80% of samples and with a relative abundance of over 0.01%. According to the criteria, a total of 778 core ASVs were identified from all samples. Interestingly, there were more core ASVs in the FA groups, which consisted of the results of alpha diversity indexes. Among these core ASVs, only 517 core ASVs overlapped with those from the TRF group (Figure 2J).

**Figure 3:**
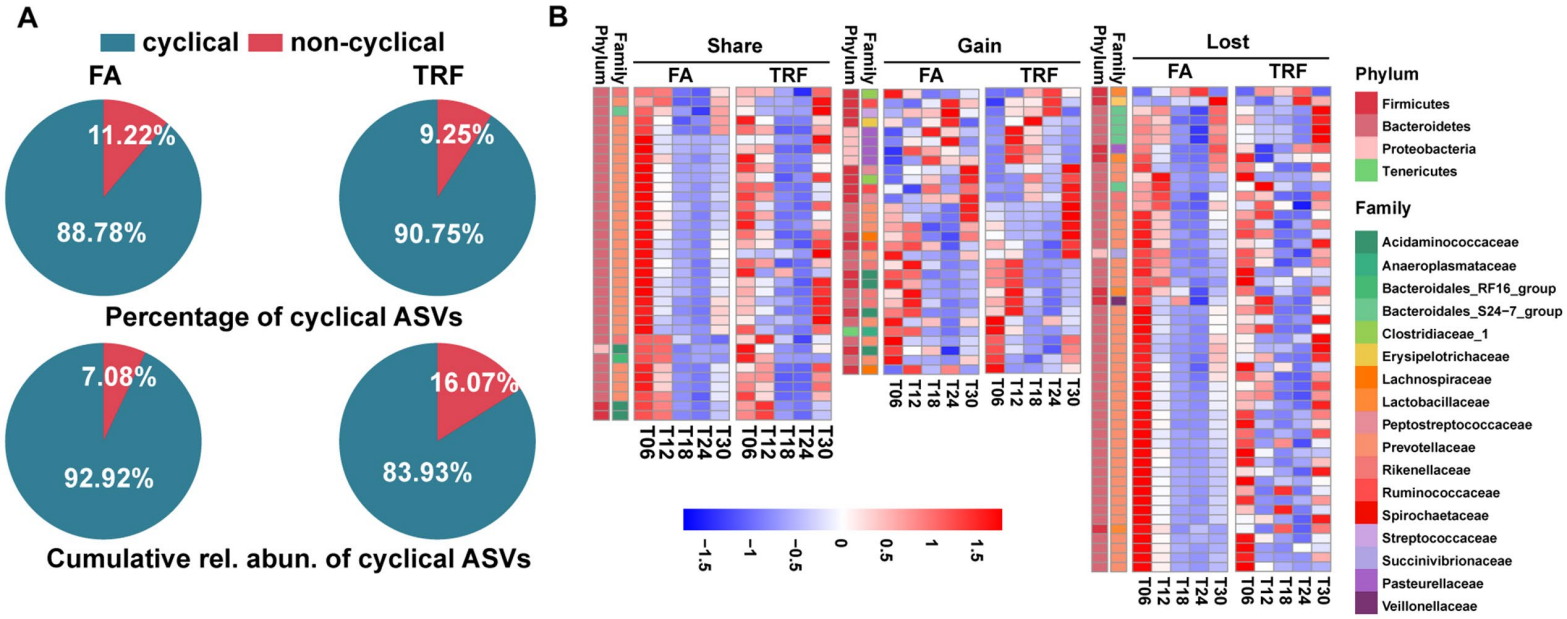
Effect of TRF on the dynamic changes of the colonic microbial community of growing pigs at the ASV level. **(A)** Piechart showing the percentage of cyclical ASVs and the accumulative relative abundance of these ASVs with rhythmicity in each group. **(B)** Heatmap depicting the effect of TRF on the dynamic fluctuations of these microbes with rhythmicity in at least one group at the ASV level. Colors represent the scaled relative abundance of each microbe with blue and red representing low and high abundance, respectively.

Bray–Curtis distance-based PCoA model separated the TRF group from the FA group clearly (Figure 2K). Further, multiple comparisons found that samples from different time points also fluctuated over time regardless of the feeding mode (Figure 2K; Supplementary Table 4). Results of rhythmicity analysis revealed that TRF decreased the percentage of cyclical ASVs while increasing the accumulative relative abundance of ASVs with rhythmicity (Figure 3A). Compared with the FA group, TRF lost the rhythmicity of *Bacteroidales*_S24−7_group ASVs, *Lachnospiraceae* ASVs, and over 50% of the cyclical *Prevotellaceae* ASVs, whereas gained the rhythmicity of *Pasteurellaceae* ASVs and *Acidaminococcaceae* ASVs. It is worth noting that ASVs that kept rhythmicity under both the FA and the TRF regimes mainly belonged to *Prevotellaceae* and *Acidaminococcaceae* (Figure 3B).

**Figure 4:**
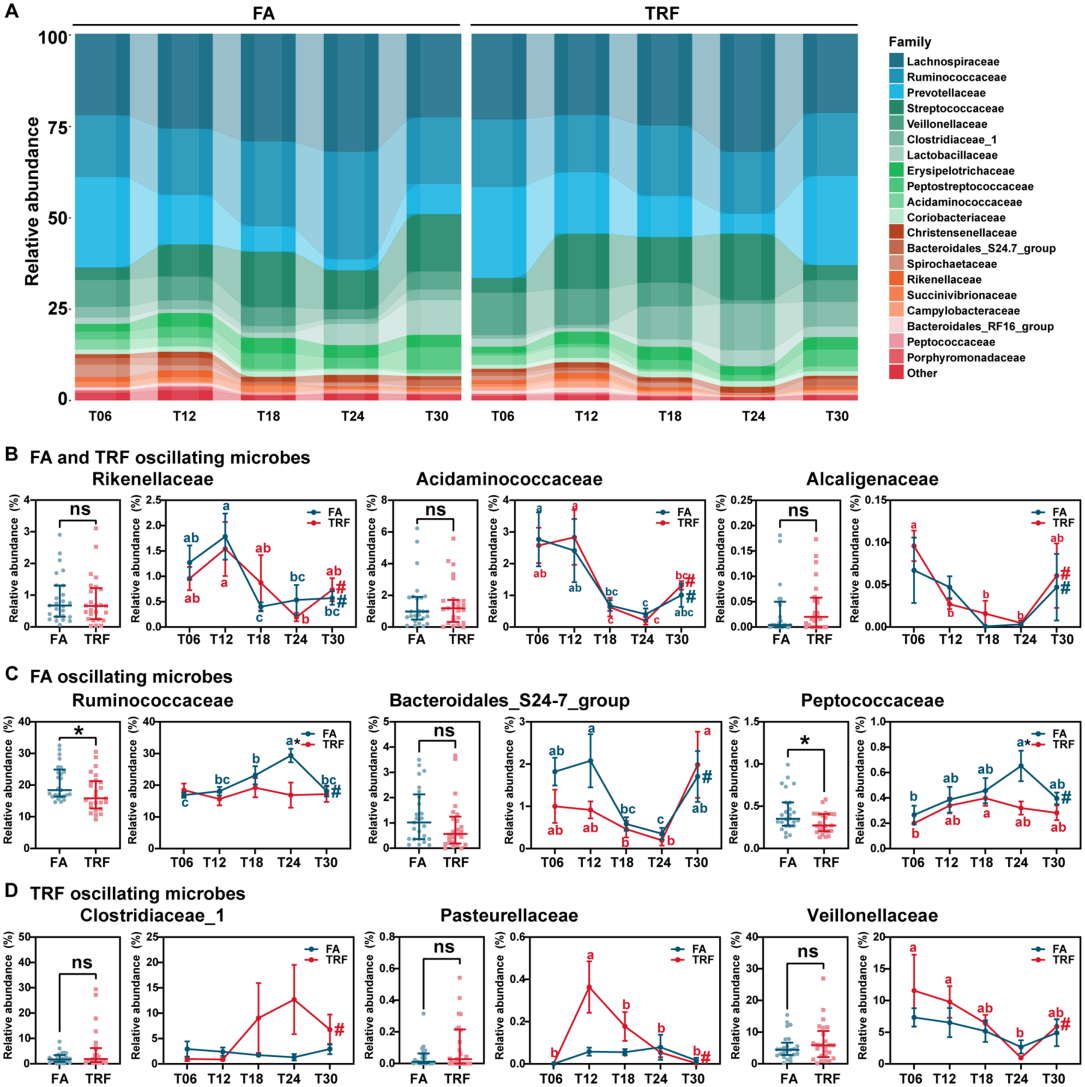
Effect of TRF on the dynamic changes of the colonic microbial community of growing pigs at the family level. **(A)** Dynamic oscillations of top 20 families in the FA and TRF groups. **(B–D)** Overall difference and dynamic fluctuation in families with rhythmicity both in the FA group and the TRF group **(B)**, only in the FA group **(C)**, and only in the TRF group **(D)**. The overall difference and the difference in the relative abundance of microbes between the FA group and the TRF group at each time point was determined by a non-parametric test. * denotes a significant difference with a *p* < 0.05 whereas ns represents *p* > 0.05. Differences in the relative abundance of microbes at the different time points of each group were identified by one-way analysis of variance, followed by Bonferroni-based multiple comparisons. Line points with different letters represent a significant difference within different time points. The rhythmicity of the relative abundance of microbes in each group was determined by JTK_circle analysis. # denotes *P*_Adj_ < 0.05.

At the family level, *Rikenellaceae* (FA: *P*_Adj_ = 0.005; TRF: *P*_Adj_ = 0.021), *Acidaminococcaceae* (FA: *P*_Adj_ = 0.012; TRF: *P*_Adj_ = 1.68 × 10^−4^), and *Alcaligenaceae* (FA: *P*_Adj_ = 0.027; TRF: *P*_Adj_ = 0.002) exhibited significant rhythmicity regardless of the feeding mode (Figures 4A,B). However, TRF shifted the trough of *Rikenellaceae* and *Alcaligenaceae* with a 6-h delay (Figure 4B). Compared with the FA group, TRF has lost the rhythmicity of *Prevotellaceae* (FA: *P*_Adj_ = 9.66 × 10^−5^; TRF: *P*_Adj_ = 0.075), *Ruminococcaceae* (FA: *P*_Adj_ = 0.004; TRF: *P*_Adj_ = 1.00), *Bacteroidales*_S24-7_group (FA: *P*_Adj_ = 0.014; TRF: *P*_Adj_ = 0.371), and *Peptococcaceae* (FA: *P*_Adj_ = 0.034; TRF: *P*_Adj_ = 0.087; Figures 4A,C) and has gained the rhythmicity of *Pasteurellaceae* (FA: *P*_Adj_ = 0.683; TRF: *P*_Adj_ = 0.001), *Clostridiaceae*_1 (FA: *P*_Adj_ = 0.003; TRF: *P*_Adj_ = 1.000), *Veillonellaceae* (FA: *P*_Adj_ = 0.101; TRF: *P*_Adj_ = 0.012), and *Peptostreptococcaceae* (FA: *P*_Adj_ = 0.795; TRF: *P*_Adj_ = 0.047; Figures 4A,D). Of note, TRF decreased the relative abundance of *Ruminococcaceae* and *Peptostreptococcaceae* at T24 when these microbes peaked in the FA group.

**Figure 5:**
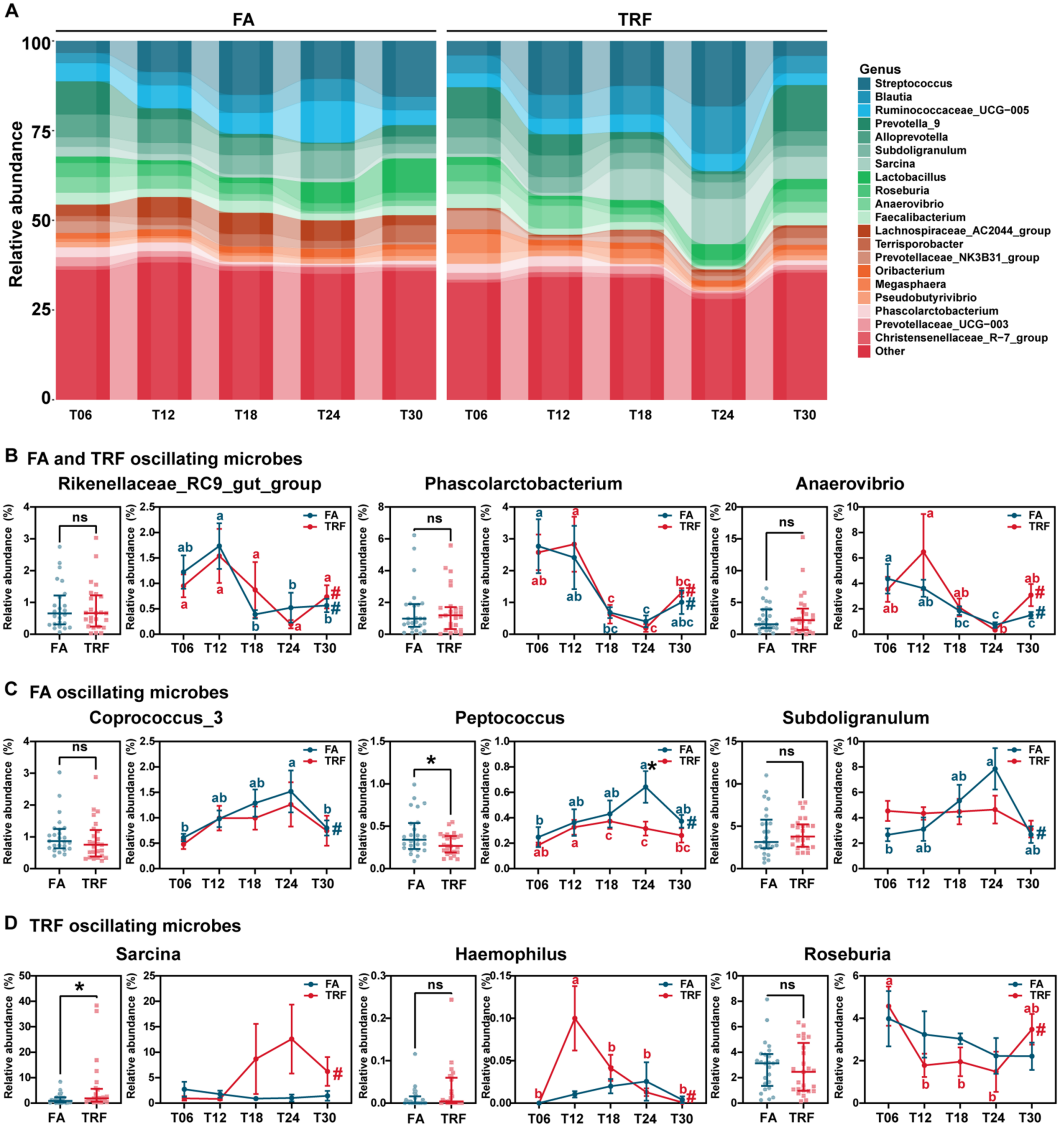
Effect of TRF on the gut of microbes in the colon community of growing pigs at the genus level. **(A)** Dynamic oscillation of top20 genera in the FA group and the TRF group. **(B–D)** Overall difference and dynamic fluctuation in genera with rhythmicity both in the FA group and the TRF group **(B)**, only in the FA group **(C)**, and only in the TRF group **(D)**. The overall difference and the difference in the relative abundance of microbes between the FA group and the TRF group at each time point was determined by an unpaired two-tailed *t*-test. * denotes a significant difference with a *p* < 0.05 whereas ns represents *p* > 0.05. Differences in the relative abundance of microbes at the different time points of each group were identified by one-way analysis of variance, followed by Bonferroni-based multiple comparisons. Line points with different letters represent a significant difference within different time points. The rhythmicity of the relative abundance of microbes in each group was determined by JTK_circle analysis. # denotes *P*_Adj_ < 0.05.

At the genus level, *Rikenellaceae_RC9_gut_group* (FA: *P*_Adj_ = 0.006; TRF: *P*_Adj_ = 0.021), *Phascolarctobacterium* (FA: *P*_Adj_ = 0.012; TRF: *P*_Adj_ = 1.68 × 10^−4^), *Anaerovibrio* (FA: *P*_Adj_ = 2.19 × 10^−4^; TRF: *P*_Adj_ = 0.012), *Sutterella* (FA: *P*_Adj_ = 0.027; TRF: *P*_Adj_ = 0.002), *Prevotella_9* (FA: *P*_Adj_ = 3.69 × 10^−4^; TRF: *P*_Adj_ = 0.008), and *Prevotella_1* (FA: *P*_Adj_ = 5.43 × 10^−5^; TRF: *P*_Adj_ = 0.040) showed significant rhythmicity both in the FA group and the TRF group (Figures 5A,B). Similarly, TRF tended to delay the peak or trough time point compared with the FA group. For example, *Rikenellaceae_RC9_gut_group* and *Sutterella* reached a trough at T18 in the FA group, whereas reached a trough at T24 in the TRF group. Compared with the FA group, TRF lost the rhythmicity of *Prevotellaceae_NK3B31_group* (FA: *P*_Adj_ = 8.17 × 10^−6^; TRF: *P*_Adj_ = 0.175), *[Ruminococcus]_gauvreauii_group* (FA: *P*_Adj_ = 0.002; TRF: *P*_Adj_ = 0.467), *Prevotellaceae_UCG-003* (FA: *P*_Adj_ = 0.002; TRF: *P*_Adj_ = 0.134), *Coprococcus_3* (FA: *P*_Adj_ = 0.012; TRF: *P*_Adj_ = 0.522), *Prevotellaceae_UCG-001* (FA: *P*_Adj_ = 0.027; TRF: *P*_Adj_ = 0.467), *Ruminococcaceae_UCG-014* (FA: *P*_Adj_ = 0.029; TRF: *P*_Adj_ = 1.00), *Subdoligranulum* (FA: *P*_Adj_ = 0.029; TRF: *P*_Adj_ = 1.00), and *Peptococcus* (FA: *P*_Adj_ = 0.047; TRF: *P*_Adj_ = 0.101; Figures 5A,C) whereas gained the rhythmicity of *Haemophilus* (FA: *P*_Adj_ = 0.120; TRF: *P*_Adj_ = 0.001), *Sarcina* (FA: *P*_Adj_ = 1.000; TRF: *P*_Adj_ = 0.006), *Actinobacillus* (FA: *P*_Adj_ = 0.683; TRF: *P*_Adj_ = 0.008), *Prevotella_2* (FA: *P*_Adj_ = 0.087; TRF: *P*_Adj_ = 0.010), *Clostridium_sensu_stricto_1* (FA: *P*_Adj_ = 1.000; TRF: *P*_Adj_ = 0.021), *Terrisporobacter* (FA: *P*_Adj_ = 0.718; TRF: *P*_Adj_ = 0.047), *Romboutsia* (FA: *P*_Adj_ = 1.000; TRF: *P*_Adj_ = 0.040), and *Roseburia* (FA: *P*_Adj_ = 1.000; TRF: *P*_Adj_ = 0.047; Figures 5A,D). Overall, TRF had limited effects on these fluctuating microbes but increased the relative abundance of *Sarcina* (*p* = 0.047) and decreased that of *Peptococcus* (*p* = 0.023).

### 3.3. TRF altered the microbial crosstalk at different sampling timepoints

**Figure 6:**
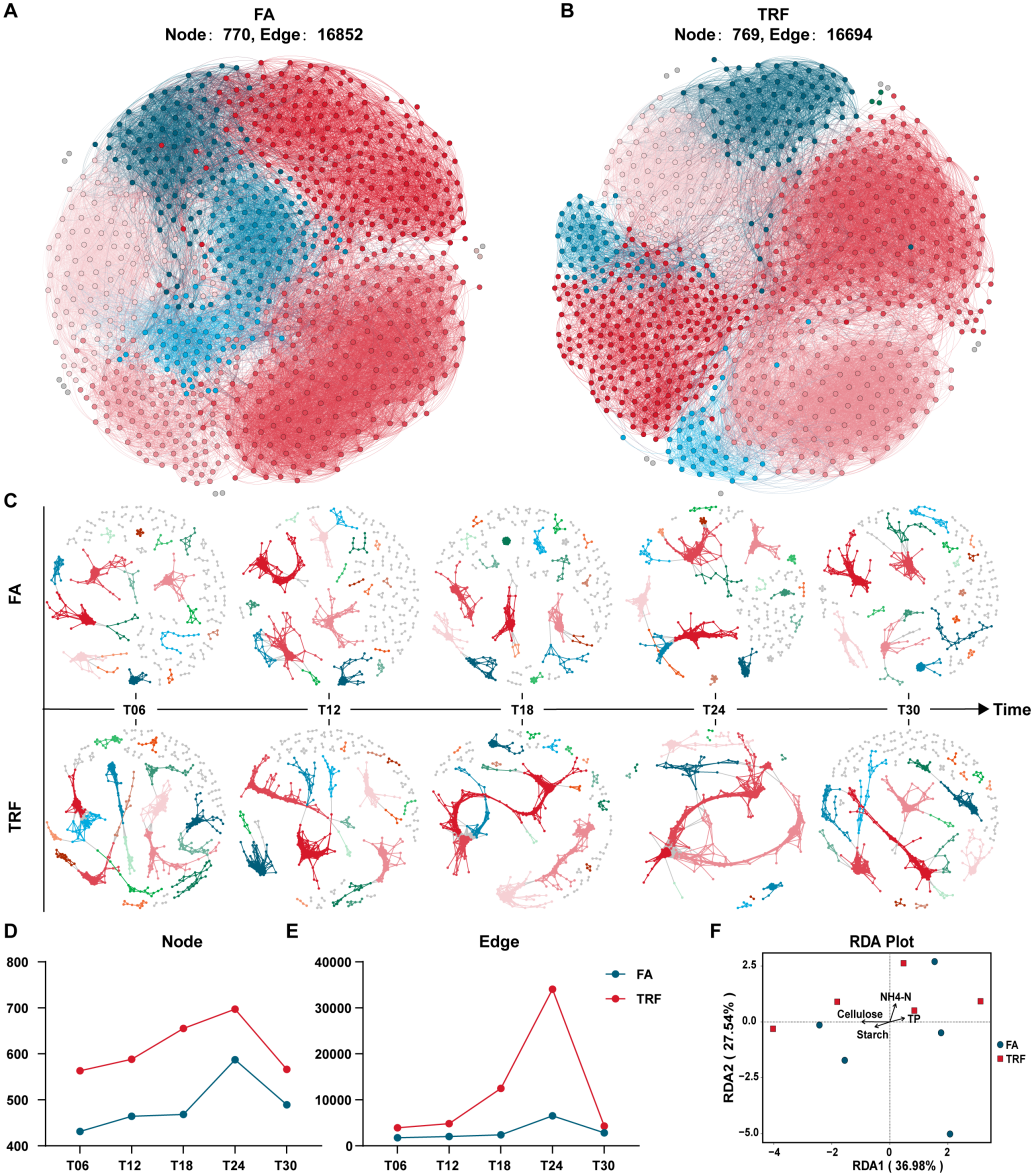
Effect of TRF on the microbial interactions in the colon of growing pigs within a day. **(A,B)** The overall microbial co-occurrence network of the FA **(A)** and the TRF **(B)** group. **(C)** Microbial co-occurrence network at different sampling time points of the FA and TRF groups. **(D,E)** Node **(D)** and edge **(E)** in the microbial co-occurrence network at different sampling time points of the FA and TRF groups. **(F)** Redundancy analysis (RDA) shows the explanation of the colonic nutrient substrates to the microbial variation at T24. NH4-N, Ammonia nitrogen; TP, total protein.

The overall microbial co-occurrence network of the FA group and TRF group had no significant difference in the populations of participant nodes and their interactions (Figures 6A,B). However, microbial co-occurrence network analysis of different sampling time points revealed that TRF had changed the interaction pattern by increasing the microbes involved in the co-occurrence network and their crosstalk, especially at T24 (Figures 6C–E). Further RDA analysis (Figure 6F; Supplementary Figure 1) implies that the microbial variation might be explained by the colonic substrates starch, cellulose, and NH4-N.

### 3.4. TRF changed the microbial metabolites in the colon of growing pigs

**Figure 7:**
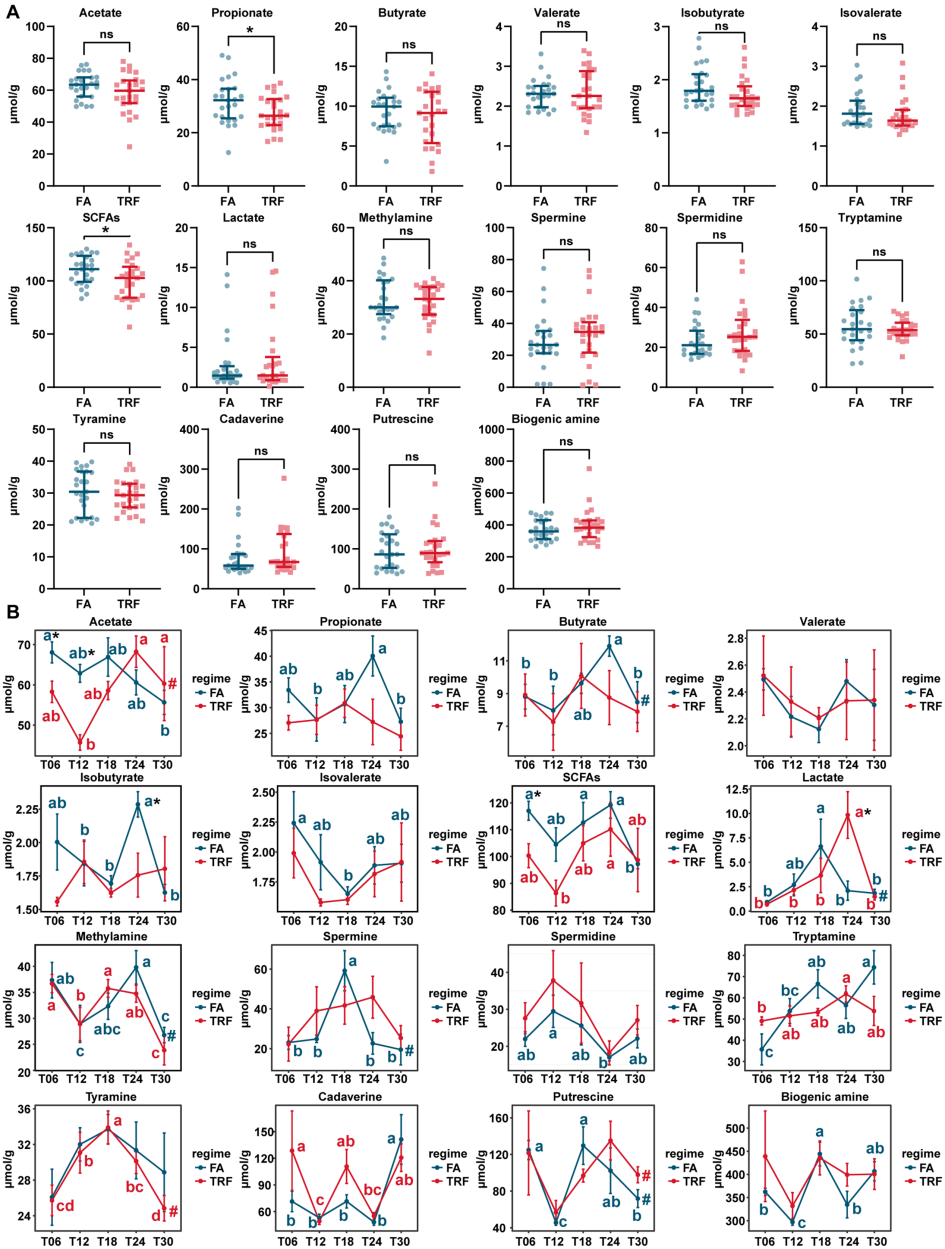
Effect of TRF on the microbial metabolites in the colon of growing pigs. **(A)** Effect of TRF on the overall concentration of the microbial metabolites in the colon. The difference between the FA group and the TRF group was determined by an unpaired two-tailed *t*-test. Ns represents *p* > 0.05, whereas * denotes a significant difference with a *p* < 0.05. **(B)** Effect of TRF on the dynamic changes in microbial metabolite acetate, propionate, butyrate, valerate, isobutyrate, isovalerate, total SCFAs, and lactate, methylamine, spermine, spermidine, tryptamine, tyramine, cadaverine, putrescine and total biogenic amine of the colonic microbial community. The difference in acetate, propionate, butyrate, valerate, total SCFAs, methylamine, tryptamine, and tyramine, between the FA group and the TRF group at each time point was determined by an unpaired two-tailed *t*-test. Whereas the differences in isobutyrate, isovalerate, lactate, spermine, spermidine, cadaverine, putrescine, and total biogenic amine between the FA group and the TRF group were determined by a non-parametric test. * denotes a significant difference with a *p* < 0.05. Differences in the relative abundance of microbial metabolites at the different time points of each group were identified by a one-way analysis of variance, followed by Bonferroni-based multiple comparisons. Line points with different letters represent a significant difference within different time points. The rhythmicity of the relative abundance of microbial metabolites of each group was determined by JTK_circle analysis. # denotes *P*_Adj_ < 0.05.

The concentration of microbial metabolites acetate, propionate, butyrate, isobutyrate, isovalerate, total SCFAs, and lactate of different time points exhibited significant differences (Figure 7B). Overall, TRF has decreased the concentration of propionate (*p* = 0.045) and the total SCFAs (*p* = 0.033; Figure 7A). During the experiment, TRF has significantly increased lactate (*p* = 0.028) and but decreased acetate at T06 (*p* = 0.030) and T12 (*p* < 0.001), isobutyrate (*p* = 0.029) at T24, and SCFA (*p* = 0.020) at T06. Although TRF has limited effects on the concentration of microbial metabolites, it has significantly changed the oscillating patterns of acetate, propionate, butyrate, isobutyrate, isovalerate, lactate, spermine, tryptamine, and putrescine (Figure 7B).

### 3.5. The difference in nutrient substrates-microbes crosstalk under different feeding mode

**Figure 8:**
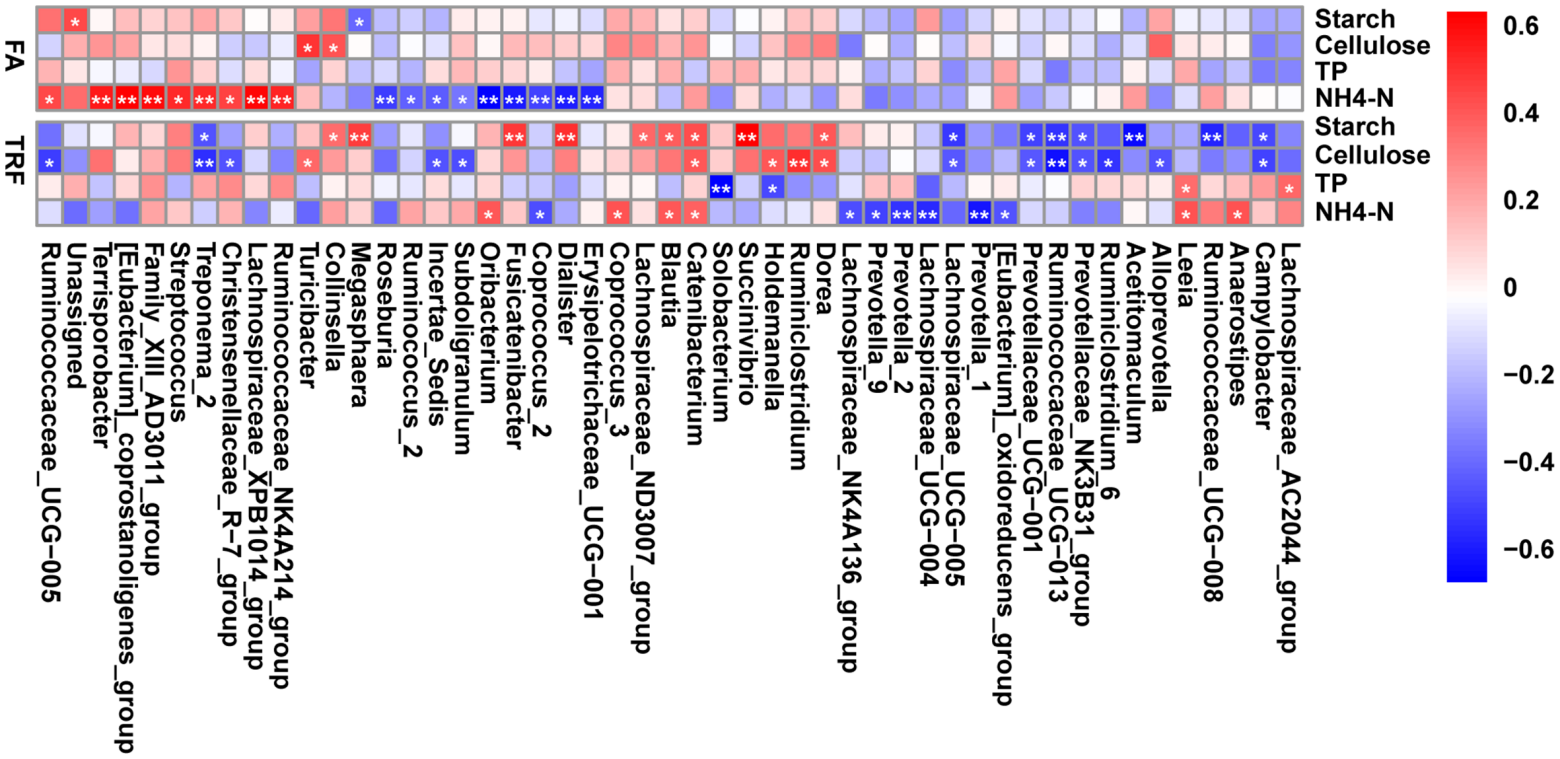
Heatmap depicting the correlation relationships between the gut microbes and the nutrients of the colonic substrate. The correlation coefficients were determined by Spearman’s correlation analysis. Correlations with red boxes indicate positive correlations and correlations with blue boxes indicate negative correlations. * denotes a significant correlation with a *P* between 0.01 and 0.05, whereas ** denotes *p* < 0.01. NH4-N, Ammonia nitrogen; TP, total protein.

Interestingly, further correlation analysis indicated that these gut microbes exhibited differential associations with the nutrient substrates under different feeding modes (Figure 8). Under the FA feeding condition, NH4-N positively correlated with *Ruminococcaceae_UCG-005* (r = 0.422, *p* = 0.037), *Ruminococcaceae_NK4A214_group* (r = 0.518, *p* = 0.009), *Terrisporobacter* (r = 0.550, *p* = 0.005), *[Eubacterium]_coprostanoligenes_group* (r = 0.620, *p* = 0.001), *Family_XIII_AD3011_group* (r = 0.583, *p* = 0.003), *Streptococcus* (r = 0.505, *p* = 0.011), *Treponema_2* (r = 0.512, *p* = 0.010), and *Lachnospiraceae_XPB1014_group* (r = 0.600, *p* = 0.002), whereas negatively correlated with *Roseburia* (r = −0.545, *p* = 0.006), *Ruminococcus_2* (r = −0.463, *p* = 0.021), *Incertae_Sedis* (r = −0.476, *p* = 0.017), *Subdoligranulum* (r = −0.398, *p* = 0.050), *Oribacterium* (r = −0.718, *p* < 0.001) *Fusicatenibacter* (r = −0.644, *p* = 0.001), *Coprococcus_2* (r = −0.538, *p* = 0.006), *Dialister* (r = −0.625, *p* = 0.001), and *Erysipelotrichaceae_UCG-001* (r = −0.612, *p* = 0.001). Meanwhile, starch and cellulose had limited associations with these microbes. Starch negatively correlated with *Megasphaera* (r = −0.439, *p* = 0.029). Cellulose was positively correlated with *Turicibacter* (r = 0.478, *p* = 0.017) and *Collinsella* (r = 0.400, *p* = 0.049). Whereas under the TRF pattern, NH4-N positively correlated with *Catenibacterium* (r = 0.422, *p* = 0.037), *Blautia* (r = 0.449, *p* = 0.025), *Oribacterium* (r = 0.452, *p* = 0.024), *Anaerostipes* (r = 0.465, *p* = 0.020), *Leeia* (r = 0.469, *p* = 0.018), and *Coprococcus_3* (r = 0.469, *p* = 0.019) whereas negatively correlated with *Prevotella*_1 (r = −0.584, *p* = 0.003), *Prevotella_2* (r = −0.514, *p* = 0.009), *Prevotella_9* (r = −0.468, *p* = 0.019), *Lachnospiraceae_UCG-004* (r = −0.532, *p* = 0.007), *Lachnospiraceae_NK4A136_group* (r = −0.444, *p* = 0.027), *Coprococcus_2* (r = −0.445, *p* = 0.027), and *[Eubacterium]_oxidoreducens_group* (r = −0.427, *p* = 0.033). Cellulose and starch had tight correlations with these microbes under the TRF pattern. Cellulose positively correlated with *Turicibacter* (r = 0.411, *p* = 0.042), *Catenibacterium* (r = 0.448, *p* = 0.026), *Holdemanella* (r = 0.460, *p* = 0.023), *Dorea* (r = 0.474, *p* = 0.018), and *Ruminiclostridium* (r = 0.560, *p* = 0.004) whereas negatively correlated with *Ruminococcaceae_UCG-013* (r = −0.601, *p* = 0.002), *Ruminiclostridium_6* (r = −0.509, *p* = 0.010), *Ruminococcaceae_UCG-005* (r = −0.488, *p* = 0.014), *Prevotellaceae_NK3B31_group* (r = −0.424, *p* = 0.035), and *Prevotellaceae_UCG-001* (r = −0.421, *p* = 0.036). Starch positively correlated with *Blautia* (r = 0.434, *p* = 0.031), *Dorea* (r = 0.449, *p* = 0.0253), *Collinsella* (r = 0.403, *p* = 0.047), *Lachnospiraceae_ND3007_group* (r = 0.408, *p* = 0.044), *Catenibacterium* (r = 0.485, *p* = 0.015), *Megasphaera* (r = 0.521, *p* = 0.008), *Fusicatenibacter* (r = 0.528, *p* = 0.007), *Dialister* (r = 0.563, *p* = 0.003), and *Succinivibrio* (r = 0.686, *p* < 0.001) whereas negatively correlated with *Ruminococcaceae_UCG-008* (r = −0.551, *p* = 0.005), *Ruminococcaceae_UCG-013* (r = −0.507, *p* = 0.010), *Prevotellaceae_UCG-001* (r = −0.472, *p* = 0.017), and *Prevotellaceae_NK3B31_group* (r = −0.434, *p* = 0.030). TP has limited effects on the gut microbiota under both feeding modes. TP was positively correlated with *Leeia* (r = 0.398, *p* = 0.049) and *Lachnospiraceae_AC2044_group* (r = 0.418, *p* = 0.038), whereas negatively correlated with *Solobacterium* (r = −0.650, *p* < 0.001) and *Holdemanella* (r = −0.455, *p* = 0.023).

## 4. Discussion

**Figure 9:**
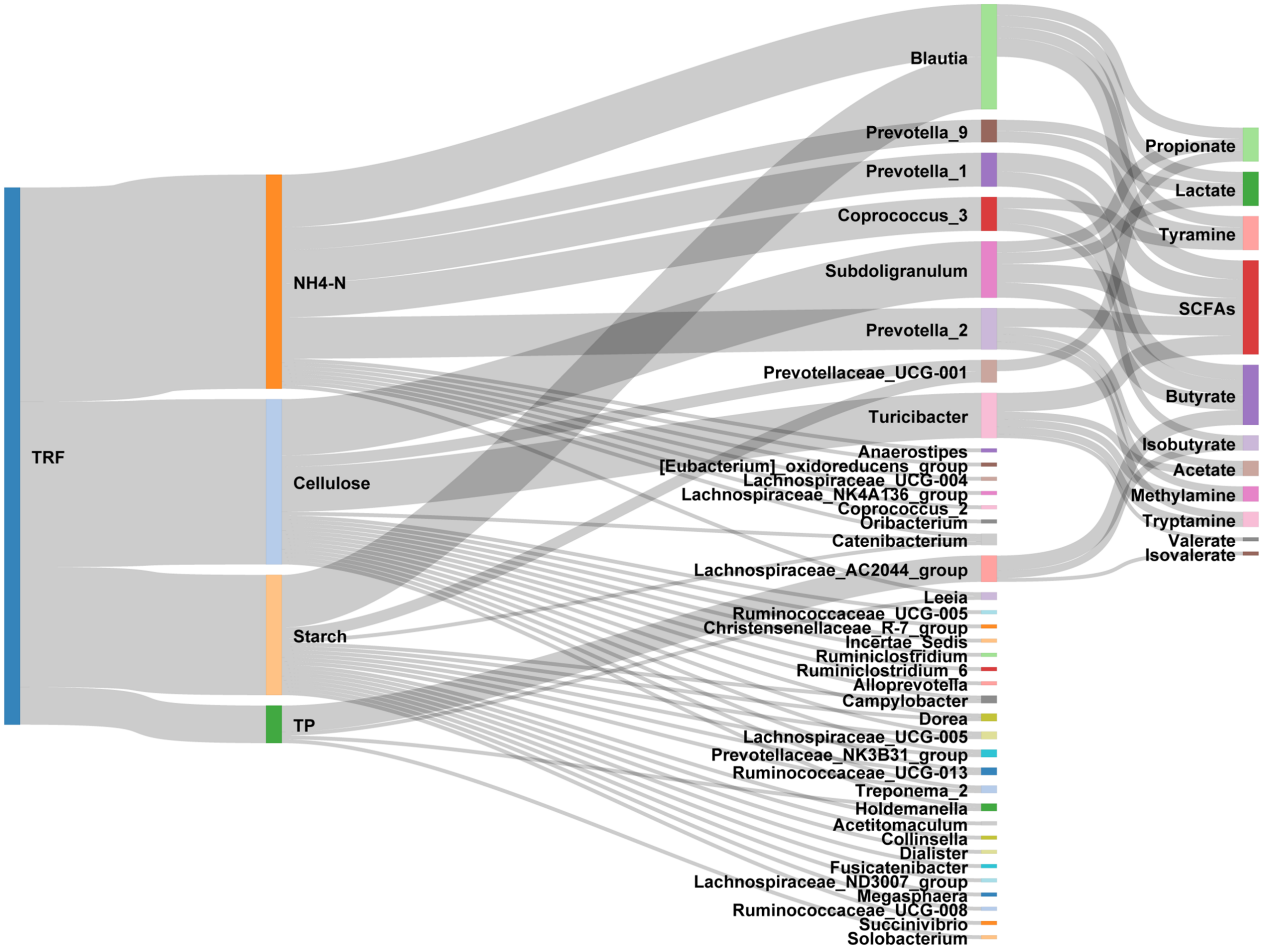
Sankey diagram exhibiting the effects of TRF on the interactions between nutrients, gut microbes, and the microbial metabolites.

In the present study, we found that TRF affected the gut microbes and their metabolites by altering the nutrient substrates therein (Figure 9). In modern society, the ever-increasing incidences of chronic metabolic diseases including obesity and diabetes have become a global public health concern. Most of these metabolic diseases have tight relationships with the misalignment of the circadian clock induced by unfavorable living habits like a high-fat diet, an irregular diet, and staying up late (Pencina et al., 2009; Shah et al., 2020). The disturbances of circadian clocks induced by nonpunctual eating exacerbated the abnormal accumulation of fat in pigs (van Erp et al., 2020). Whereas TRF can effectively decrease body weight gain, reduce fat accumulation, and improve metabolic health (Sherman et al., 2011; Hatori et al., 2012; Sherman et al., 2012). Our previous research reveals that TRF has increased average daily body weight gain without changing the average daily feed intake (Xia et al., 2022). TRF has gradually become an effective therapy for the treatment and prevention of obesity and other metabolic diseases. However, the mechanisms by which TRF exerts its effects are currently inconclusive.

Convincing evidence indicated that TRF could entrain the periodic clock and thus affect the hormone-secretion and gene expression in the colonic mucosa (Boumans et al., 2017; Zeb et al., 2020a; Ye et al., 2021). Interestingly, research revealed that TRF altered the dynamic metabolism of lipids and amino acids without affecting the host circadian clock (Lundell et al., 2020) and restored the loss of the gut rhythmicity induced by the misalignment of the circadian clock. Therefore, the effects of TRF on the circadian clock could be exerted through the gut microbiota (Zeb et al., 2020a; Daas and de Roos, 2021). TRF has extensively influenced microbial diversity, gut microbiota composition, and microbial metabolites (Zheng et al., 2020; Zeb et al., 2020b; Wang et al., 2021). Xie et al. (2022) found that the alpha-diversity index was increased by a mid-day TRF pattern. In the present study, we found that TRF has decreased the alpha-diversity Richness, chao1, and ACE at T12, whereas it increased Invsimpson at T30. These results indicated that the effects of TRF at each time point were not always consistent. This result is a reminder that we should attach importance to the sampling time when conducting animal trials, especially targeting parameters related to the gut microbiota, host physiology, and body metabolism, which exhibit robust diurnal fluctuation. Compared with these references, the study emphasized the effects of TRF on the dynamic fluctuations of gut microbiota and the crosstalk between the colonic nutrient substrates and microbiota. As nutrients from the feed in the fecal or distal colon digesta were mostly metabolized. Thus, we choose digesta from the proximal colon to conduct this study based on fistula pig model. In the present study, we found that TRF has significantly decreased the concentration of colonic cellulose. There are two possible explanations for the observation. Relevant studies have indicated that changing the feeding frequency or altering the feeding pattern could alter the digestibility of nutrients in the feed including the fiber digestibility (Jia et al., 2021; Xia et al., 2022). Colonic nutrient substrates in samples from fecal or distal colon digesta were mostly metabolized. Besides, samples from distal colon digesta are too hard to pass through the cannula. Thus, proximal colonic digesta were used in the present study. In addition, we found that the crosstalk among these microbes was more active in the TRF groups, which might imply a more powerful metabolic capability of cellulose. Furthermore, our findings indicated that cellulose had more associations with these microbes under the TRF feeding mode. Increasing evidence suggests that the alterations in the gut microbiota induced by TRF are tightly associated with nutrients (Zeb et al., 2020b; Wang et al., 2021). We found that despite its limited effects on the gut microbiota at each time point, TRF extensively altered their dynamic fluctuations within a day.

Further, the colonic nutrients were tightly correlated with the gut microbe and explained most microbial variation. Numerous studies demonstrated that nutrients are determinant in shaping gut microbiota composition (Sun et al., 2015, 2016; Liu et al., 2018; Yu et al., 2019). Undigested carbohydrates (mainly cellulose and starch), which escaped digestion in the small intestine, can selectively stimulate the proliferation of specific microbes and thus alter the microbial composition in the large intestine (Yang et al., 2013). A high-cellulose diet has increased the relative abundance of *Oscillibacter*, whereas it decreased the relative abundance of *Akkermansia* in mice (Kim et al., 2020). In addition, adding 10% dietary cellulose to the feed of chickens increased the relative abundance of Peptococcaceae, Peptostreptococcaceae, and *Eubacterium* (Cao et al., 2003). Consistently, our results also found that the cellulose concentration was positively correlated with the *Peptococcaceae* genus including *Peptococcus*, *Peptostreptococcaceae*, and *Terrisporobacter*. But what should be taken into consideration is that both the source, linkage type, chain length, particle size, anomers, and epimers of dietary fiber might affect the fiber-microbiota interaction (Hamaker and Tuncil, 2014).

On the other hand, TRF is a broad concept that different patterns will lead to considerably different effects. For example, compared with the 12 equal meals/day group, the two equal meals/day group decreased lipid accumulation and improved the inflammation response (Yan et al., 2020). Fed with the same high-fat diet, an 8-h TRF significantly reduced the total energy intake and increased the gut hormones Ghrelin and Adiponectin in the serum compared with the 12-h TRF group (Sundaram and Yan, 2016). Within 12 h TRF patterns, feeding in the light phase has gained 13% more body weight than feeding in the night phase (Salgado-Delgado et al., 2010). Of note, the effect of TRF also depended on age (Chaix et al., 2021). Despite that it is hard to compare the age stage of a pig and a human, the gut microbial function of adult humans is comparable to that of pigs in growing stage (Xiao et al., 2016). These findings together indicated that the effects of TRF are affected by many factors. Therefore, individualized treatment therapy should be developed to meet the needs of specific clinical circumstances.

SCFAs, especially acetic, propionic, and butyric acid, are vital to maintaining normal biological rhythms of the host (Segers et al., 2019, 2020). In this work, we found that the cycling behaviors of these SCFAs could be regulated by the TRF feeding pattern. A possible explanation is that TRF changed the dynamics of key nutrients and consequentially changed the composition and the relative abundance of the microbes, thus the microbial metabolites. Our work provides new insight into modulating host metabolism and the gut microbiota through shifting the eating/feeding habits. However, as a preliminary attempt, the precise mechanism of how these cycling microbes are associated with these SCFAs could not be clearly elucidated yet. We need further in-depth studies to address these issues.

## Data availability statement

The datasets presented in this study can be found in online repositories. The names of the repository/repositories and accession number(s) can be found in the article/Supplementary material.

## Ethics statement

The animal study was reviewed and approved by the Animal Care and Use Committee of Nanjing Agricultural University (Nanjing, Jiangsu Province, China; SYXK2019-0066).

## Author contributions

WZ: conceptualization, supervision, and writing—review and editing. YS: project administration, funding acquisition, conceptualization, supervision, writing—review and editing, and methodology. HW: conceptualization, formal analysis, investigation, writing—original draft, and visualization. QL: methodology, investigation, validation, and writing—reviewing and editing. RX: methodology, validation, and writing—reviewing and editing. All authors contributed to the article and approved the submitted version.

## Funding

This work was supported by grants from the National Natural Science Foundation of China (32072688 and 31872362).

## Conflict of interest

The authors declare that the research was conducted in the absence of any commercial or financial relationships that could be construed as a potential conflict of interest.

## Publisher’s note

All claims expressed in this article are solely those of the authors and do not necessarily represent those of their affiliated organizations, or those of the publisher, the editors and the reviewers. Any product that may be evaluated in this article, or claim that may be made by its manufacturer, is not guaranteed or endorsed by the publisher.
