# Supplementary figures and images for: Time-restricted feeding affects colonic nutrient substrates and modulates the diurnal fluctuation of microbiota in pigs
Source: Front Microbiol. 2023 May 19;14:1162482. doi: 10.3389/fmicb.2023.1162482 (PMC10235616; doi:10.3389/fmicb.2023.1162482)

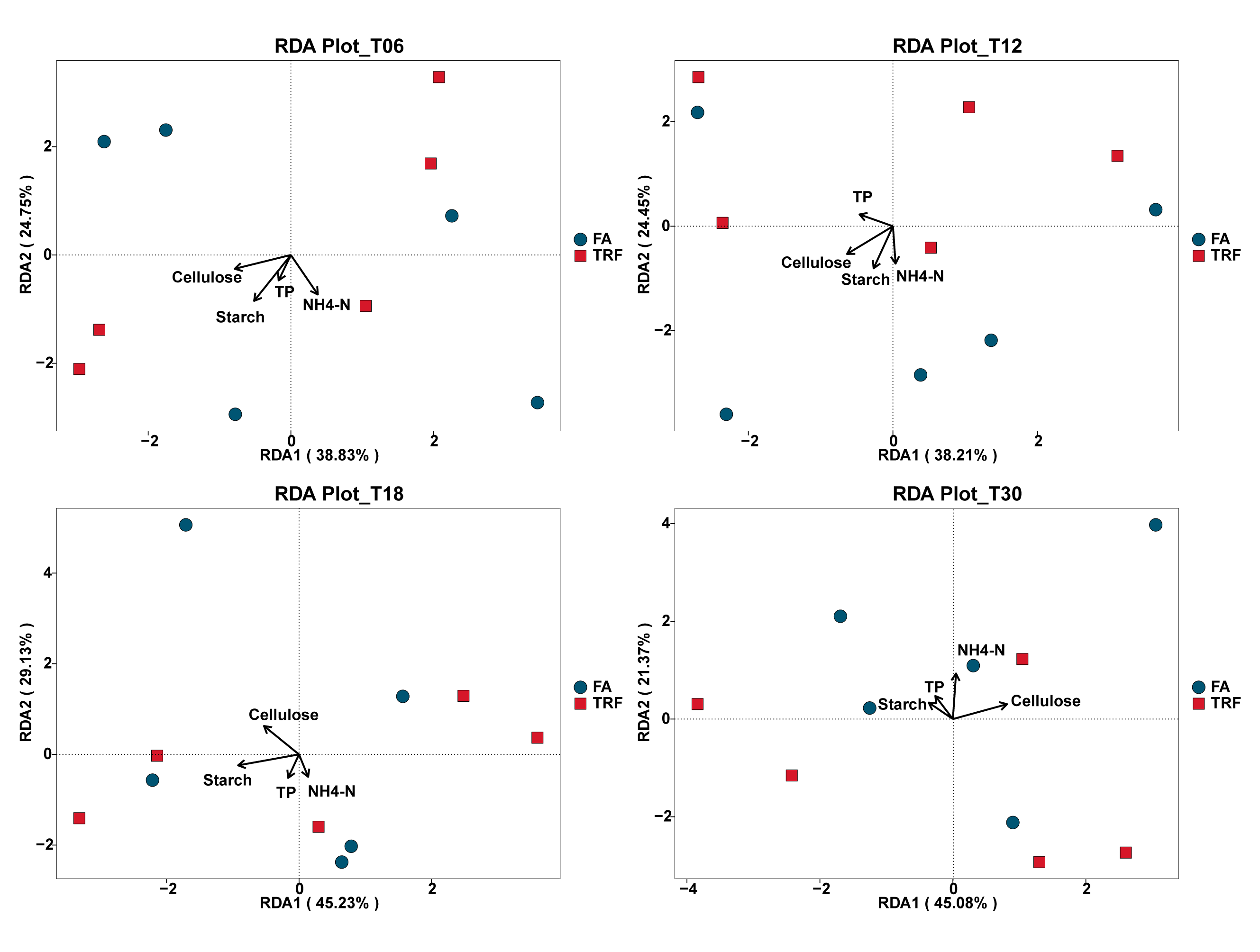

Supplement: Supplementary file 5 [file Image_1.TIF]
